# Supplementary material for: Performance of the FebriDx Rapid Point-of-Care Test for Differentiating Bacterial and Viral Respiratory Tract Infections in Patients with a Suspected Respiratory Tract Infection in the Emergency Department
Source: J Clin Med. 2023 Dec 27;13(1):163. doi: 10.3390/jcm13010163 (PMC10779507; doi:10.3390/jcm13010163)
Supplement: Supplementary file 1 [file jcm-13-00163-s001.zip › jcm-2733063-supplementary.pdf]

**Table S1:** criteria used for clinical adjudication.

Clinical adjudication criteria. The expert panel used the available clinical data to determine the infection status. PCT values were used in the patients without a confirmed or ruled out infection. The expert panel was strongly recommended to classify patients with a PCT >0.25 ng/mL as likely bacterial infection and patients with a PCT<0.10 ng/mL as unlikely infection. However, if the other clinical data evidently suggested another infection category, the expert panel was allowed to discard the PCT recommendation.

| **Bacterial infection** | | | | | |
|---|---|---|---|---|---|
| | **Criteria** | | **Examples of findings** | | |
| **Adjudication** | **Definition** | **Cultures and molecular testing** | **Clinical data** | **Laboratory tests** | **Imaging** |
| *Confirmed* | Positive cultures or molecular testing with bacterial pathogen related to clinical infection. | Positive and clinically plausible, non-colonizer, non-contaminant | Cough with colored sputum, crepitation on auscultation.<br>Evidence of pus drainage from a normally sterile site during surgery or intervention | PCT > 0.25 ng/mL | Conventional, CT (Computed Tomography) or ultrasound imaging confirming infection |
| *Likely* | Bacterial infection likely, but not confirmed by cultures. | Negative or suggestive but unconfirmatory of infection. | Cough with colored sputum, crepitation on auscultation. | PCT > 0.25 ng/mL | Conventional, CT or ultrasound imaging suggestive of infection |
| *Unlikely* | Bacterial infection unlikely but possible without an alternative non-bacterial diagnosis | Negative | Mild or non-specific symptoms | PCT<0.1 ng/mL | No suggestive findings of bacterial infection |
| No infection | Bacterial infection unlikely with an alternative non-bacterial diagnosis | Negative | Mild or non-specific symptoms | PCT<0.1 ng/mL | No suggestive findings of bacterial infection |
| **Viral infections** | | | | | |

| Adjudication | Criteria | | Examples of findings | | |
|---|---|---|---|---|---|
| | **Definition** | **Cultures and molecular testing** | **Clinical data** | **Laboratory tests** | **Imaging** |
| *Confirmed* | Positive molecular or serology testing on virus related to clinical infection | Positive PCR or IgM positive in serology testing. | Clinical signs and symptoms confirming of viral pathogen, such as fever and muscle ache | No specific abnormalities | CT imaging confirmatory of infection |
| *Likely* | Viral infection likely but unconfirmed by molecular or serology testing | Negative or suggestive but unconfirmatory of infection. | Clinical signs and symptoms confirming of viral pathogen, such as fever and muscle ache. At risk infection by SARS-CoV-2 by positive tested friends or family in the past 5 days | No specific abnormalities | CT imaging suggestive of infection |
| *Unlikely* | Viral infection unlikely, but no alternative non-viral diagnosis | Negative | Mild or non-specific symptoms | No specific abnormalities | No suggestive findings of viral infection |
| No infection | Viral infection unlikely with an alternative non-viral diagnosis | Negative | Mild or non-specific symptoms | No specific abnormalities | No suggestive findings of viral infection |

**Table S2.** Overview of missing data per variable. CRP: C-reactive protein.

| Variable | Number of patients missing | Percentag e |
|---|---|---|
| Heart rate | 3 | 1,2 |
| Systolic blood pressure | 2 | 0,8 |
| Diastolic blood pressure | 3 | 1,2 |
| Temperature | 2 | 0,8 |
| Respiratory rate | 2 | 0,8 |
| CRP | 2 | 0,8 |
| Procalcitonin | 5 | 2,0 |
